# Derivation and validation of a prognostic score for neonatal mortality in Ethiopia: a case-control study

**DOI:** 10.1186/s12887-020-02107-8

**Published:** 2020-05-20

**Authors:** Rishi P. Mediratta, Ashenafi Tazebew Amare, Rasika Behl, Bradley Efron, Balasubramanian Narasimhan, Alemayehu Teklu, Abdulkadir Shehibo, Mulugeta Ayalew, Saraswati Kache

**Affiliations:** 1 Department of Pediatrics, Stanford University School of Medicine, Stanford, California USA; 2 Department of Pediatrics and Child Health, University of Gondar, College of Medicine and Health Sciences, Gondar, Ethiopia; 3 Department of Biomedical Data Science, Stanford University, Stanford, California USA; 4 Department of Pediatrics, Stanford University School of Medicine, Division of Critical Care, Stanford, California USA

**Keywords:** Neonatal early warning score, Neonatal scoring systems, Neonatal mortality, Newborns, Ethiopia, Neonatal intensive care unit

## Abstract

**Background:**

Early warning scores for neonatal mortality have not been designed for low income countries. We developed and validated a score to predict mortality upon admission to a NICU in Ethiopia.

**Methods:**

We conducted a retrospective case-control study at the University of Gondar Hospital, Gondar, Ethiopia. Neonates hospitalized in the NICU between January 1, 2016 to June 31, 2017. Cases were neonates who died and controls were neonates who survived.

**Results:**

Univariate logistic regression identified variables associated with mortality. The final model was developed with stepwise logistic regression. We created the Neonatal Mortality Score, which ranged from 0 to 52, from the model’s coefficients. Bootstrap analysis internally validated the model. The discrimination and calibration were calculated. In the derivation dataset, there were 207 cases and 605 controls. Variables associated with mortality were admission level of consciousness, admission respiratory distress, gestational age, and birthweight. The AUC for neonatal mortality using these variables in aggregate was 0.88 (95% CI 0.85–0.91). The model achieved excellent discrimination (bias-corrected AUC) under internal validation. Using a cut-off of 12, the sensitivity and specificity of the Neonatal Mortality Score was 81 and 80%, respectively. The AUC for the Neonatal Mortality Score was 0.88 (95% CI 0.85–0.91), with similar bias-corrected AUC. In the validation dataset, there were 124 cases and 122 controls, the final model and the Neonatal Mortality Score had similar discrimination and calibration.

**Conclusions:**

We developed, internally validated, and externally validated a score that predicts neonatal mortality upon NICU admission with excellent discrimination and calibration.

## Introduction

In 2017 alone, 2.5 million neonates died globally, with almost 80% deaths occurring in sub-Saharan African and Southern Asia [1]. Between 2000 and 2017, although overall under-five mortality decreased, the proportion of global neonatal deaths among under-five children increased from 40 to 47% [1]. In particular, Ethiopia ranks as having the 21st worst neonatal mortality rate, with 29 deaths per 1000 live births in 2017 [1]. Four out of every fifth neonatal death could be prevented with simple tools [2]. Despite the introduction of neonatal intensive care units (NICUs), neonatal deaths remain high in low- and middle- countries (LMICs). Low-resource NICUs are often unable to provide simple life-sustaining medical intervention due to a lack of trained health personnel, equipment deficiencies, and drug shortages [3].

One strategy to improve the early identification of patients at risk of dying is to develop and implement early warning scores in hospitals [4]. Early warning scores assign a number to physiologic parameters in order to derive a composite score that identifies patients who need additional interventions and monitoring. Studies have demonstrated the efficacy of early warning scores in adult and pediatric patient populations [5–7]. However, there are no validated neonatal mortality prediction tools for LMICs. Prognostic scores have been proposed in neonates [8–19], but all include laboratory tests that are generally not available in low-resource settings, include ventilator support metrics, and require trained providers for scoring.

To date, no early warning score for neonatal mortality has been derived and validated for NICUs in low-resource settings. Creation of such a score for LMICs would allow over-burdened health care personnel to rapidly identify at-risk neonates. The aim of the project is to derive and validate an admission prognostic score using easily measurable and accessible variables for neonates admitted to a NICU in Ethiopia.

## Methods

### Study design, data source, and patient selection

This Neonatal Mortality Score was derived and validated from a retrospective, case-control study at the University of Gondar Hospital in Gondar, Ethiopia, a teaching hospital located approximately 700 km from the capital city of Addis Ababa. This hospital serves more than 7 million individuals and cares for approximately 10,000 children every year. The hospital is staffed by sixth-year medical students, pediatric residents, and general practitioners. The NICU in Gondar has approximately 40 beds in which neonates can receive thermoregulation, nasogastric tube feedings, phototherapy, blood transfusions, intravenous fluids, antibiotics, oxygen via nasal canula, and bubble continuous positive airway pressure (CPAP). The NICU admission criteria include the following: birthweight less than 2000 g, gestational age less than 34 weeks, suspected or confirmed infection, temperature instability, respiratory distress, apnea, cyanosis, electrolyte derangements, birth trauma, seizures, birth asphyxia, altered mentation, feeding problem, bilious emesis, signs of bowel obstruction, hyperbilirubinemia, ABO and Rh incompatibility, anemia, polycythemia, bleeding disorder, cardiovascular disease requiring monitoring or interventions, any baby whom the physician or nurse feels the baby requires observation or treatment, and social issues like abandoned babies. The unit does not have a neonatologist and does not have mechanical ventilation capabilities; however, there is a plan to start mechanical ventilation and procure an arterial blood gas machine in the near future. The challenges in Gondar are similar to other NICUs in developing countries with limited resources, technology, and personnel [20].

Cases were defined as newborns who died in the NICU, and controls were defined as newborns who survived. In the derivation and external validation datasets, patients were recruited from the NICU registry. The derivation dataset consisted of newborns admitted from January 1, 2016 to December 31, 2016, and the external validation dataset consisted of newborns admitted from January 1, 2017 to June 31, 2017. Cases and controls were recruited sequentially. Patients older than 28 days and outside of the accrual period were excluded. Data abstracters were not blind to the predictors or outcome.

### Predictor variables

The following predictor variables were extracted into REDCap based on review of the literature and biological plausibility: diagnosis on admission, maternal age, age of baby, gender, gestational age, type of delivery, duration of labor, duration of rupture of membranes, APGAR scores, birth weight, head circumference, and length at admission. Gestational age was determined by the New Ballard score. Clinical values included admission heart rate, respiratory rate, temperature, mental status, and respiratory distress. Admission mental status and respiratory distress were abstracted from the initial physical exam recorded by the clinicians.

Initial vital signs upon NICU admission were categorized according to World Health Organization definitions [21, 22]. Temperature in Celsius was categorized as normal from 36.5 to 37.5, cold stress from 36.0 to 36.4, hypothermia below 36.0, and fever above 37.5 [21]. Normal heart rate was defined as 100 to 160 beats per minute, bradycardia less than 100 beats per minute, and tachycardia above 160 beats per minute. Respiratory rate was defined as bradypnea less than 30 breaths per minute, normal respiratory rate was defined as 30 to 60 breaths per minute, and tachypnea was above 60 breaths per minute. Low birth weight was defined less than 2500 g and very low birth weight was defined less than 1500 g [22]. Respiratory distress was categorized as none; mild distress had subcostal and intercostal retractions; moderate distress had subcostal, intercostal, nasal flaring, and grunting; severe distress had subcostal, intercostal, nasal flaring, grunting, and perioral cyanosis.

Small-for-gestational age (SGA), appropriate-for-gestational age (AGA), large-for-gestational age (LGA), microcephalic, normocephalic, and macrocephalic were defined according to the reference distributions [23]. SGA was defined as birthweight below the 10th percentile for gestational age, AGA was defined as birthweight between the 10th and 90th percentiles for gestational age, and LGA was defined as birthweight above the 90th percentile for gestational age. Microcephalic was defined as head circumferences below the 10th percentile for gestational age, normocephalic was defined as head circumference between the 10th and 90th percentiles for gestational age, and macrocephalic was defined as head circumference greater than 90th percentile for gestational age.

### Outcome variable

The dependent variable was neonatal mortality in the NICU.

### Sample size

No prior estimates were available to calculate the sample size for the derivation study. Hence, the rule of thumb of 10 events per variable for logistic regression prediction models was used to estimate the sample size [24]. Since there were 20 candidate variables considered and 10 events per variable, the estimated number of cases for the derivation study was 200.

### Missing data

Prediction variables missing 15% or more of data were excluded from the analysis. We imputed missing values with the mode for categorical data or the median for continuous data.

### Statistical analysis

#### Model derivation

We conducted univariate logistic regression on the derivation dataset to investigate the relationship between each predictor and NICU mortality. Statistically significant variables (*p* <  0.05) from the univariate analysis were entered into a backward stepwise multivariate logistic regression model, and significant variables (p <  0.05) were retained in the multivariate model. Since all NICU admissions from 2016 were included, three times as many cases were identified as controls. Each case was weighted three times that of one control. The results of significant predictors were reported as coefficients, odds ratios (ORs), and 95% confidence intervals (CI).

#### Model performance

The discrimination was assessed by calculating the C-statistic, the area under the ROC curve (AUC), sensitivity, and specificity. Calibration plots of observed and predicted probabilities of mortality, the calibration intercept and slope, and the Hosmer-Lemeshow goodness of fit statistic were generated. Internal validation of the model was conducted on the derivation cohort using bootstrap sampling. Bias-corrected mean and 95% CIs of the C-statistic, sensitivity, and specificity were calculated by bootstrapping 2000 samples with replacement. Bootstrapping with replacement mimics randomly sampling from the population [25].

#### External validation

The external validity of the model was assessed by applying the multivariate coefficients from the derivation dataset to data from a different time period at the same hospital. We calculated the calibration and discrimination of both the multivariate model and the Neonatal Mortality Score in the validation dataset.

#### Developing the neonatal mortality score

In order to create a clinically useful and accurate Neonatal Mortality Score, the regression coefficients from the final multivariate model were used to assign integers to each variable based on a method by Sullivan et al. [26]. The score was internally validated using bootstrap sampling. The cut-off area was defined as having 50% probability of mortality.

Data were analyzed using Stata 15 (College Station, TX). Two-sided *P* values less than 0.05 defined statistical significance. Descriptive analyses were performed between the derivation and validation group using the χ^2^ test (categorical variable) or Student’s t-test (continuous variable). The Transparent Reporting of a Multivariable Prediction Model for Individual Prognosis or Diagnosis checklist was followed [27].

#### Sensitivity analyses

First, we assessed the extent to which neonates who died within 4 h of admission influenced the overall model. Neonates who immediately died were omitted from the derivation dataset and the multivariable analysis was repeated. Second, we assessed the extent to which missing data from the 5-min APGAR score influenced the results. Complete case analysis was performed in order to examine the extent to which the 5-min APGAR score influenced the final model.

## Results

### Descriptive analyses

The derivation dataset contained 812 patients, comprising 207 cases and 605 controls, and the validation dataset contained 246 patients, composed of 124 cases and 122 controls. For unclear reasons, there were approximately three times as many controls as cases in the derivation dataset and approximately equal numbers of cases and controls in the validation dataset. Among the newborns in the derivation dataset, 66% were term and 60% were males. Among newborns in the validation dataset, 61% were term gestational age and 59% were males. The demographic characteristics for the both datasets are displayed in Table 1. There were fewer neonates in the validation dataset, primarily because of the shorter period of recruitment in the validation dataset. The derivation dataset differed from the validation dataset with regard to prematurity, low birth weight, respiratory distress, altered mental status, bradycardia, and bradypnea. These clinical differences explain the greater observed mortality rate in the validation dataset as compared to the derivation dataset. The following variables were not missing data: gestational age, admission heart rate, admission respiratory rate, admission temperature, admission respiratory distress, admission altered mental status, type of delivery, birthweight, and CPAP use on admission. There were no participants in either dataset missing the final outcome. The following variables in the derivation dataset had more than 15% missing data and were excluded from the multivariate analysis: duration of labor, rupture of membranes, 1st minute APGAR, and 5th minute APGAR.

**Table 1:** Characteristics of Neonatal Intensive Care Unit Patients for Derivation and Validation Datasets

	No. (%)		
**Baseline Characteristics**	**Derivation Set (*n*** **= 812)**	**Validation Set (*n*** **= 246)**	**p**
Time, year	2016	2017	
Mortality	207 (26%)	124 (50%)	0.41
Maternal Age, year			0.72
< 20	54 (8%)	19 (8%)	
21–29	452 (65%)	150 (66%)	
≥ 30	186 (27%)	59 (26%)	
Parity			0.30
1	409 (51%)	132 (54%)	
≥ 2	400 (49%)	111 (46%)	
Admission Age, hour			0.05
≤ 1	431 (53%)	113 (46%)	
> 1	381 (47%)	133 (54%)	
Sex			0.75
Male	488 (60%)	145 (59%)	
Female	325 (40%)	101 (41%)	
Gestational Age, weeks			0.25
≥ 37 weeks	532 (66%)	150 (61%)	
32–36 weeks	193 (24%)	56 (23%)	
< 32 weeks	87 (11%)	40 (16%)	
Birthweight, grams			0.03
≥ 2500	465 (57%)	127 (52%)	
1500–2499	263 (32%)	77 (31%)	
< 1500	84 (10%)	42 (17%)	
Onset of Labor			0.09
Spontaneous	666 (94%)	205 (91%)	
Induced	40 (6%)	20 (9%)	
Duration of labor, mean (SD), hours	11.8 (9.7)	11.9 (10.8)	0.84
Rupture of membranes, mean (SD), hours	9.8 (31.2)	19.2 (77.6)	0.02
Delivery			0.03
Vaginal	572 (70%)	191 (78%)	
C-Section	240 (30%)	55 (22%)	
Antenatal Care	764 (94%)	234 (95%)	0.68
Maternal HIV positive	29 (4%)	10 (4%)	0.77
1st Minute APGAR, mean (SD)	6.5 (1.5)	5.9 (1.8)	< 0.001
5th Minute APGAR, mean (SD)	7.7 (1.5)	7.2 (1.7)	< 0.001
Suctioned at Delivery	161 (20%)	60 (24%)	0.12
Bag & Mask at Delivery	138 (17%)	65 (26%)	< 0.001
Intubated at Delivery	22 (3%)	3 (1%)	0.18
CPAP used on Admission	181 (22%)	83 (34%)	< 0.001
Admission Heart Rate			< 0.001
< 100	14 (2%)	20 (8%)	
100–160	714 (88%)	202 (82%)	
> 160	84 (10%)	24 (10%)	
Admission Respiratory Rate			0.24
< 30	28 (3%)	25 (10%)	
30–60	481 (59%)	128 (52%)	
> 60	303 (37%)	93 (38%)	
Admission Temperature, Celsius			0.38
< 36.0	525 (65%)	157 (64%)	
36.0–36.4	90 (11%)	25 (10%)	
36.5–37.5	139 (17%)	40 (16%)	
> 37.5	58 (7%)	24 (10%)	
Admission Respiratory Distress			0.0047
None	396 (49%)	96 (39%)	
Mild	64 (8%)	20 (8%)	
Moderate	233 (29%)	82 (33%)	
Severe	119 (15%)	48 (20%)	
Admission Level of Consciousness			< 0.001
Alert	677 (84%)	167 (68%)	
Irritable	21 (3%)	4 (2%)	
Lethargic	91 (11%)	53 (22%)	
Comatose	23 (3%)	22 (9%)	
Gestational Size			0.50
Small for Gestational Age	263 (32%)	85 (35%)	
Appropriate for Gestational Age	502 (62%)	149 (61%)	
Large for Gestational Age	45 (6%)	12 (5%)	
Head Size			0.84
Microcephalic	51 (7%)	16 (7%)	
Normocephalic	447 (61%)	149 (63%)	
Macrocephalic	229 (31%)	72 (30%)	

APGAR = Appearance, Pulse, Grimace, Activity, and Respiration, CPAP = Continuous Positive Airway Pressure

Comparison of sociodemographic and clinical variables between derivation and validation datasets. Percentages may not add to 100% due to rounding, and numbers may not add to the total due to missing values

### Derivation and internal validation

The univariate analysis of the derivation dataset is displayed in Table 2. The following variables were associated with NICU mortality: gestational age, birthweight, suctioned at delivery, bag mask ventilation at delivery, intubated at delivery, CPAP on admission, admission heart rate, admission respiratory rate, admission temperature, admission respiratory distress, and admission altered mental status. We sought to derive a model that reflected the clinical presentation of neonates prior to interventions in the NICU, therefore CPAP on admission was not included in the multivariate analysis.

**Table 2:** Univariate Analysis from the Derivation Dataset

Characteristic	Cases (%) (***n*** = 207)	Controls (%) (***n*** = 605)	OR	95% CI	p
Maternal age, years					0.05
< 20	11%	7%	1.92	1.06–3.48	
21–29	59%	68%	1		
≥ 30	30%	26%	1.37	0.93–2.01	
Parity					0.05
1	45%	53%	1		
≥ 2	55%	47%	1.37	1.0–1.89	
Admission Age, hours					0.07
≤ 1	58%	51%	1		
> 1	42%	49%	0.75	0.54–1.03	
Gender					0.79
Male	61%	60%	1		
Female	39%	40%	0.96	0.69–1.32	
Gestational Age, weeks					< 0.001
≥ 37	42%	74%	1		
32–36	25%	24%	1.86	1.26–2.79	
< 32	34%	3%	21.4	12.0–38.1	
Birthweight, grams					< 0.001
≥ 2500	34%	65%	1		
1500–2499	35%	32%	2.13	1.47–3.08	
< 1500	31%	3%	19.3	10.9–34.2	
Onset of Labor					0.11
Spontaneous	97%	94%	1		
Induced	3%	6%	0.51	0.21–1.23	
Delivery					0.07
Vaginal	75%	69%	1		
C-section	25%	31%	0.72	0.50–1.03	
Antenatal Care	92%	95%	0.54	0.29–1.00	0.06
Maternal HIV positive	4%	4%	1.18	0.51–2.71	0.70
Suctioned at Delivery	27%	17%	1.77	1.21–2.56	0.003
Bag & Mask at Delivery	28%	13%	2.46	1.67–3.61	< 0.001
Intubated at Delivery	5%	2%	2.51	1.07–5.90	0.04
CPAP on admission	59%	10%	13.8	9.37–20.34	< 0.001
Admission Heart Rate					< 0.001
< 100	5%	0.005%	12.0	3.31–45.6	
100–160	81%	90%	1		
> 160	14%	9%	1.73	1.07–2.80	
Admission Respiratory Rate					< 0.001
< 30	10%	1%	9.06	3.88–21.2	
30–60	50%	62%	1		
> 60	40%	36%	1.37	0.98–1.91	
Admission Temperature, Celsius					< 0.001
< 36.0	83%	58%	7.10	3.53–14.29	
36.0–36.4	6%	13%	2.22	0.90–5.51	
36.5–37.5	4%	21%	1		
> 37.5	6%	7%	4.17	1.67–10.42	
Admission Respiratory Distress					< 0.001
None	15%	61%	1		
Mild	5%	9%	2.53	1.20–5.35	
Moderate	40%	25%	6.75	4.27–10.7	
Severe	40%	6%	28.1	16.4–48.3	
Admission Level of Consciousness					< 0.001
Alert	59%	92%	1		
Irritable	4%	2%	3.38	1.39–8.19	
Lethargic	27%	6%	7.21	4.53–11.5	
Comatose	9%	1%	21.4	7.15–64.0	
Gestational Size					0.86
Small for Gestational Age	32%	33%	1		
Appropriate for Gestational Age	62%	62%	1.01	0.72–1.43	
Large for Gestational Age	6%	5%	1.21	0.61–2.45	
Head Size					0.65
Microcephalic	8%	6%	1		
Normocephalic	59%	62%	0.77	0.41–1.47	
Macrocephalic	33%	31%	0.87	0.45–1.70	

OR = odds ratio

Each row represents a separate univariate model. The following variables had more than 15% missing and were excluded from the multivariate analysis: duration of labor, rupture of membranes, 1st minute APGAR, and 5th minute APGAR. Percentages may not add to 100% due to rounding

**Fig. 1:**
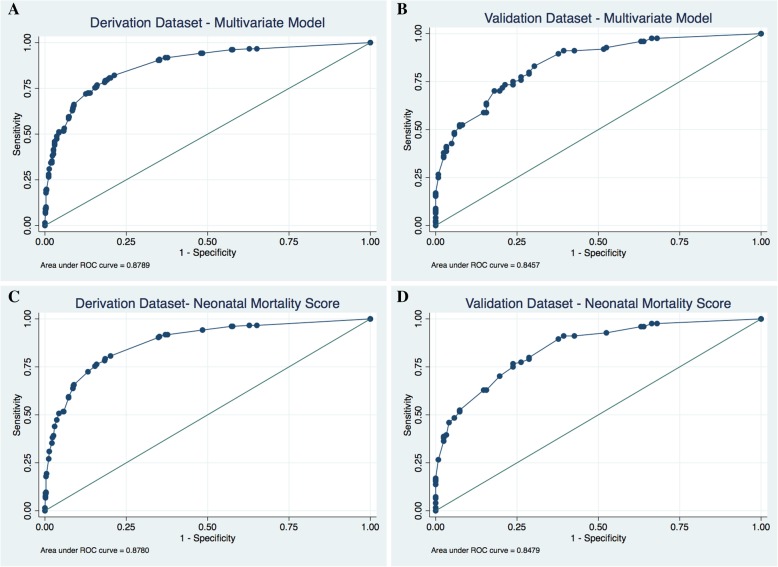
Receiver Operating Curves for Derivation and Validation Datasets. The area under the curve for the derivation and validation datasets for both the final multivariate model and the Neonatal Mortality Score

**Fig. 2:**
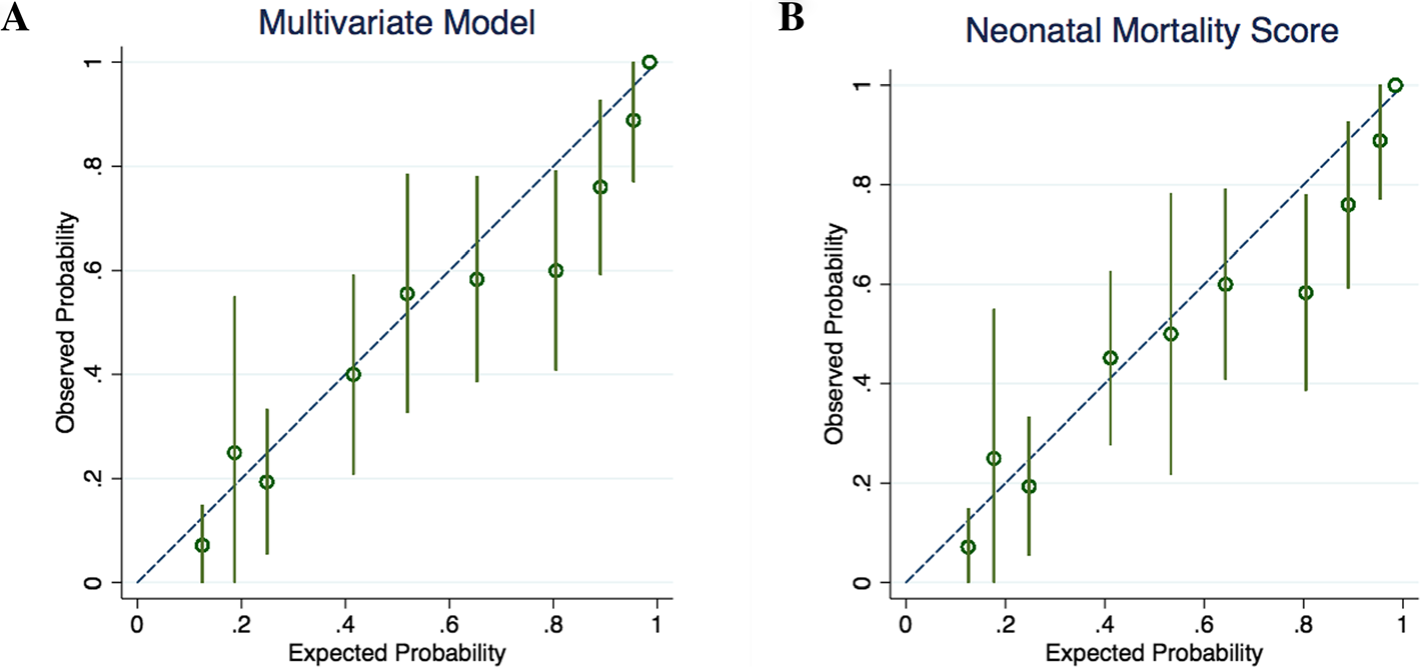
Calibration Plots of Validation Datasets. Calibration plots demonstrating observed versus expected probability of neonatal intensive care unit mortality in the derivation dataset from the multivariate model and the Neonatal Mortality Score. Error bars for 95% CI for the expected probabilities are displayed

Results of the multivariate analysis are shown in Table 3. Admission altered mental status, admission respiratory distress, gestational age, and birthweight were retained in the final model. The discriminatory power of the model was excellent since the AUC was 0.88 (95% CI 0.85–0.91) (Fig. 1). Using a predicated probability of mortality greater than 50%, the sensitivity of this model was 79%, the specificity was 82%, the positive predictive value was 85%, and the negative predictive value was 74%. After bootstrap internal validation, optimism-corrected AUC was 0.86 (95% CI 0.83–0.89). Model optimism was estimated as 0.02 indicating minimal overfitting of the model to the data. Calibration of the model was visually accurate since observed and predicted probabilities were similar, as shown in Fig. 2. The slope of the calibration plot was 0.995, indicating close agreement between observed and predicted probabilities of mortality. The calibration-in-the-large statistic was − 0.004, suggesting low systemic overprediction or underprediction. Among the 207 neonates who died in the derivation dataset, there were 37 (17%) who died immediately within 4 h of admission; in a sensitivity analysis excluding these neonates, there was no change in the discrimination of the model (AUC 0.86, 95% CI 0.83–0.90). When complete case analysis was performed in a sensitivity analysis, including the 5-min APGAR score in the final model did not change the discrimination of the model (AUC 0.90, 95% CI 0.88–0.93).

**Table 3:** Multivariate Analysis from Derivation Dataset and Neonatal Mortality Score upon Admission to the Neonatal Intensive Care Unit

Characteristic	ß coefficient	OR	95% CI	p	Score^**a**^
Admission Level of Consciousness					
Alert	0	1	Reference	Reference	0
Irritable	0.92	2.51	1.16–5.43	0.02	6
Lethargic	1.77	5.87	3.82–9.02	< 0.001	11
Comatose	2.61	13.7	4.72–39.7	< 0.001	16
Admission Respiratory Distress					
None	0	1	Reference	Reference	0
Mild	0.54	1.72	1.01–2.93	0.046	3
Moderate	1.70	5.49	4.00–7.55	< 0.001	11
Severe	2.23	9.30	5.89–14.7	< 0.001	14
Gestational Age, weeks					
≥ 37	0	1	Reference	Reference	0
32–36	0.16	1.17	0.80–1.72	0.41	1
< 32	1.63	5.12	2.63–9.97	< 0.001	10
Birthweight, grams					
≥ 2500	0	1	Reference	Reference	0
1500–2499	0.77	2.16	1.50–3.10	0.01	5
< 1500	1.89	6.61	3.39–12.9	< 0.001	12

OR = odds ratio, Intercept −1.95, ^a^ Score ranges from 0 to 52

Final multivariate model and points associated with the Neonatal Mortality Score

### External validation

The discriminatory power of the final model in the validation dataset was excellent since the area under the receiver operating characteristics curve was 0.85 (95% CI 0.80–0.89). The slope of the calibration plot for the validation dataset was 0.84, and the Hosmer-Lemeshow statistic was 16.5 (*p* = 0.09), indicating fair calibration in the external validation dataset.

### Neonatal mortality score

**Fig. 3:**
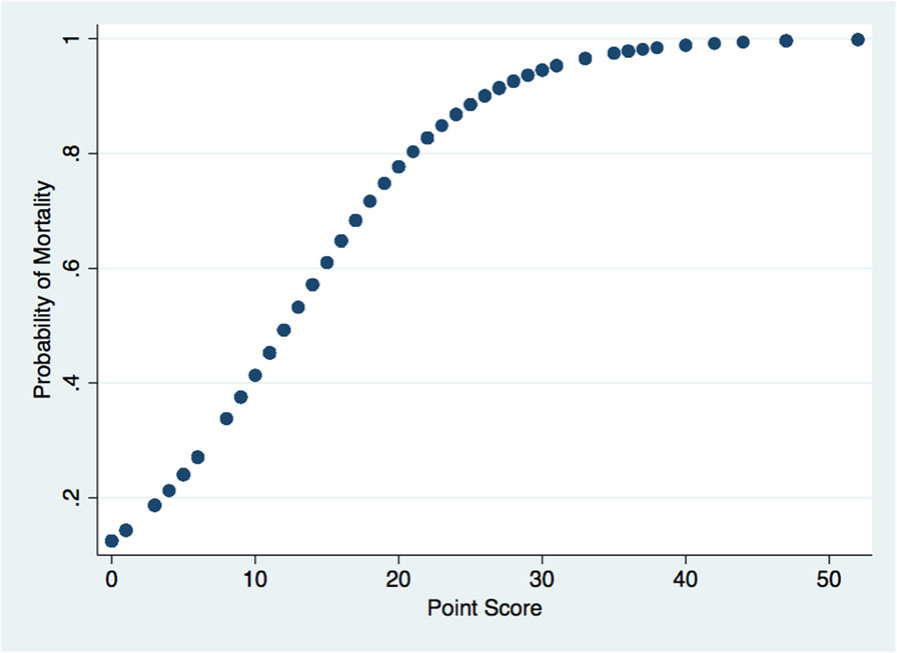
Neonatal Mortality Score. The graph shows the probability of neonatal intensive care unit mortality at different point levels

The Neonatal Mortality Score predicts neonatal mortality upon NICU admission. Each variable in the model was assigned a point value from 0 to 16 based on ß coefficients in the multivariate model (Table 1). As shown in Fig. 3, the predicted probability of NICU mortality ranged from 4% for patients with 0 points to 100% for patients with 52 points. The cut-off value for the Neonatal Mortality Score corresponding to 50% probability of mortality was 12. For this cut-off, sensitivity was 81%, specificity was 80%, positive predictive value was 58%, negative predictive value was 83%, and AUC was 0.88 (95% CI 0.85–0.91) (Fig. 1) with the derivation dataset. Bootstrap sampling revealed the bias-corrected AUC was 0.85 (95% CI 0.82–0.89). Calibration of the Neonatal Mortality Score in the derivation dataset was good since the calibration slope was 0.84 and the Hosmer-Lemeshow statistic was 16.5 (*p* = 0.09). In the validation dataset, the Neonatal Mortality Score’s discrimination was excellent since the AUC was 0.85 (95% CI 0.80–0.89). Calibration of the Neonatal Mortality Score in the validation dataset was similar to the multivariate model; the calibration slope was 0.85 and the Hosmer-Lemeshow statistic was 17.0 (*p* = 0.07).

## Discussion

We have developed and validated a Neonatal Mortality Score, a simple clinical decision tool that uses four variables for predicting neonatal mortality upon admission in one hospital’s NICU in Ethiopia. Based on the excellent discrimination and calibration both datasets, the Neonatal Mortality Score is a promising tool. We identified admission level of consciousness and respiratory distress, birthweight, and gestational age as predictors of mortality. While the Neonatal Mortality Score predicted 58% of deaths in this validation dataset, it has an excellent negative predictive value and specificity, suggesting it can be a useful initial screening tool upon admission for neonatal mortality.

This is the first study that develops and validates an early warning score for neonatal mortality in a LMIC. Prior studies have been limited to high-resource NICUs and include laboratory data as part of the mortality score, such as CRIB-II. We identified admission altered mental status and respiratory distress as new risk factors for neonatal mortality, whose strength of association in the Neonatal Mortality Score were stronger than low birthweight and prematurity– known risk factors for neonatal mortality [28, 29].

This Neonatal Mortality Score is created from individual clinical parameters that are easily accessible by frontline providers [30, 31], suggesting that the tool may be applied to clinical practice in other NICUs in LMIC settings. This integer score, which will facilitate easy implementation in the field, produces results with similar accuracy as the multivariable regression coefficients. Moreover, the study analyzed multiple maternal and neonatal variables and the derivation set had a large sample size. The study was conducted in a NICU with comparable resources and personnel to many NICUs in LMIC, so the results may be generalizable to similar resource-constrained settings [20].

The Neonatal Mortality Score may be utilized by bedside nurses and clinicians in understaffed NICUs in low resource settings to quickly identify sick neonates needing additional interventions. These results provide an opportunity to improve the identification of neonates at risk of dying, guide triage decisions within and between NICUs, and allow for appropriate allocation of personnel resources. Furthermore, neonates identified from the score may benefit from a prioritized bundle of interventions that are part of NICU care: correcting hypothermia by rewarming neonates, assessment of point-of-care glucose, insertion of an IV for parenteral fluids or antibiotics, and bubble-CPAP for respiratory distress. Moreover, the score may help frontline providers caring for neonates to identify when consultation with senior physicians may be essential.

Sepsis, a leading cause of neonatal mortality globally, often presents with respiratory distress and/or altered mental status, along with other physiologic abnormalities. In LMICs, there are barriers in obtaining supporting laboratory data for sepsis. The Neonatal Mortality Score may result in a paradigm shift of identifying neonatal sepsis without laboratory evaluation prior to the development of severe sepsis and septic shock.

A nurse in this setting will easily be caring for 5–20 patients in any given shift. The nurse often relies on the clinical exam of direct observation and the measured vital signs, but no continuous monitors. Therefore, having a score that allows rapid assessment of the neonates to identify the babies at risk of mortality with only four parameters can prove to be an incredible tool at the bedside. Once identified, the at risk neonate can quickly receive the required interventions. Moreover, such score can also allow for appropriation of limited devices such a bubble-CPAP to be used only on those patients that require it. The score may help prioritize the neonates needing limited resources the most.

Study limitations included the following. First, selection bias could be introduced by not randomizing the selection of controls. Second, our study could not assess if duration of rupture of membranes and APGAR scores influenced neonatal mortality because these variables had more than 15% missing data and were excluded from the multivariate analysis. Since APGAR was excluded, our score may not capture mortality associated from perinatal asphyxia. However, including the 5-min APGAR score in a sensitivity analysis did not meaningfully change the model. Neonates with low APGAR scores at birth likely had altered mental status and were still captured in the model. Third, altered mental status and respiratory distress are subject to varying interpretations based on the experience, clinical training, and physical exam skills of the examiner. Fourth, this retrospective study was conducted at a single institution and may not be widely generalizable. Fifth, data abstractors were not blind to the predictors and outcome, which could introduce a biased estimation of the predictors for mortality. Lastly, the sample size of the validation set is relatively small.

Further research is needed to validate the Neonatal Mortality Score in other institutions in low resource settings. Prospective validation studies will also be critical. Neonatal scoring tools that prognostically assess the risk of neonatal mortality after birth in LMICs should remain a priority.

## Conclusions

Taken together, in a single neonatal intensive care unit in Ethiopia, four variables – respiratory distress, altered mental status, birthweight, and gestational age – contributed to the Neonatal Mortality Score. The score has excellent discrimination and calibration and is a validated tool to predict neonatal mortality. We anticipate this tool will be useful for risk stratifying and guiding decisions about resource allocations and treatment upon NICU admission.

## Data Availability

Data are not available due to privacy concerns.

## Abbreviations

AGA: Appropriate-for-gestational age
AUC: Area under the ROC curve
CI: Confidence intervals
CPAP: Continuous positive airway pressure
LGA: Large-for-gestational age
LMICs: Low- and middle-income countries
NICU: Neonatal intensive care unit
OR: Odds ratio
SGA: Small-for-gestational age
